# Supplementary material for: Urban human settlements’ resilience measurement and characteristics and their mechanism model in China
Source: PLoS One. 2023 Aug 7;18(8):e0289754. doi: 10.1371/journal.pone.0289754 (PMC10406327; doi:10.1371/journal.pone.0289754)
Supplement: Attachment — Submitted filename: Response to Reviewers.docx [file pone.0289754.s002.docx]

Dear Editor and Reviewer,

Thank you for your letter. We have studied comments carefully and have made a correction which we hope meet with approval. We used "Track Changes" in the paper. The main corrections in the paper and the responses to your comments are as following:

**Qunxi Gong**

**Academic Editor :**

Please review your reference list to ensure that it is complete and correct. If you have cited papers that have been retracted, please include the rationale for doing so in the manuscript text, or remove these references and replace them with relevant current references. Any changes to the reference list should be mentioned in the rebuttal letter that accompanies your revised manuscript. If you need to cite a retracted article, indicate the article’s retracted status in the References list and also include a citation and full reference for the retraction notice.

Response:

Thank you for your suggestion. We checked our references. We replaced references [18][44][54], added reference [32] according to reviewer's comments, and removed two duplicate references.

**Reviewer #2:**

1. In the fourth paragraph of the introduction, "Firstly, from a research perspective ......" is too wordy in its explanation.

Response:

Thanks for pointing it out. We have reduced this sentence.

2. It is recommended to refer to this study in the third paragraph of the introduction：Spatiotemporal change of ecologic environment quality and human interaction factors in three gorges ecologic economic corridor, based on RSEI.

Response:

Thanks for your suggestion, we have added the reference.

3. Figure 1 is not clear and beautiful enough. And the area map should contain as much information as possible, such as elevation and topography, depending on the situation.

Response:

Thanks for the suggestion.We have modified Figure 1. However, since the research mainly focuses on urban human settlements, and Hong Kong, Macao and Taiwan are not in the research area due to data reasons, layers such as elevation and slope are not superimposed during the mapping, but a point layer is added to represent Municipalities and provincial capitals.

4. In the Index selection section, does the all-positive indicator from the Criterion layer have an impact on the results?

Response:

Thank you for your suggestion, but our indicator layer is not all positive indicators, it contains 11 negative indicators.

5. The font of Figure 2 needs to be bolded. Please note the adjustment of other unclear figures.

Response:

Thank you for your suggestion. We have made the font in Figure 2 bold.

6. In the section on Temporal and spatial characteristics of urban human settlements, the reason for the higher resilience score of the southeastern coastal provinces is not clearly stated, and the logic is not clear.

Response:

Thanks for the suggestion. We have added reasons in this section. The details are as follows:

The southeastern coastal provinces have developed economy, rapid urbanization and industrial development, and local measures to cope with pressure are adequate, thereby promoting the improvement of resilience of urban human settlements.

Thank you once again for the opportunity to comment on this manuscript. The paper has improved much and is moving towards a stronger contribution. Best wishes.

Yours sincerely,

Rongjun Ao

Professor of Key Laboratory for Geographical Process Analysis & Simulation Hubei Province, Central China Normal University
