# Supplementary material for: Urban human settlements’ resilience measurement and characteristics and their mechanism model in China
Source: PLoS One. 2023 Aug 7;18(8):e0289754. doi: 10.1371/journal.pone.0289754 (PMC10406327; doi:10.1371/journal.pone.0289754)
Supplement: Attachment — Submitted filename: Response to Reviewers.docx [file pone.0289754.s003.docx]

Dear Editor and Reviewer,

Thank you for your letter. We have studied comments carefully and have made a correction which we hope meet with approval. The main corrections in the paper and the responses to your comments are as following:

**Qunxi Gong**

**Academic Editor :**

Please review your reference list to ensure that it is complete and correct. If you have cited papers that have been retracted, please include the rationale for doing so in the manuscript text, or remove these references and replace them with relevant current references. Any changes to the reference list should be mentioned in the rebuttal letter that accompanies your revised manuscript. If you need to cite a retracted article, indicate the article’s retracted status in the References list and also include a citation and full reference for the retraction notice.

Response:

Thanks very much for your kind work. And we have checked our reference list to make sure it is complete and correct.

**Reviewer #2:**

1. Figures 3 and 4, as well as the other figures, can be resized for a more aesthetically pleasing font. Please double-check similar issues in the other figures and make minor modifications..

Response:

Thanks for pointing it out. According to Figure 1 and Figure 2 after the previous round of adjustment, we adjusted the font size in Figure 3, Figure 4, Figure 5 and Figure 6 from 6 to 7.5. The fonts in Figures 5 and 6 have also been bolded.

Thank you once again. The paper has improved much and is moving towards a stronger contribution. Best wishes.

Yours sincerely,

Rongjun Ao

Professor of Key Laboratory for Geographical Process Analysis & Simulation Hubei Province, Central China Normal University
